# CREATING AN AGE-SAVVY WORKFORCE THROUGH GERONTOLOGICAL INTERNSHIPS

**DOI:** 10.1093/geroni/igad104.0486

**Published:** 2023-12-21

**Authors:** Rona Karasik, Laura Donorfio, Marilyn Gugliucci

## Abstract

The disconnect between age and work demographics is well-known. Less understood is how to create an age-savvy workforce prepared to meet the diverse needs of a rapidly aging population. To date, considerable research has focused on: (1) Are current and future professionals interested in working with older adults (e.g., King et al., 2013) (2) Does “exposing” current and future professionals to aging content increase their interest in working with older adults (e.g., Gutheil, et al, 2009); and (3) What impact do direct intergenerational experiences have on student attitudes and desires to work with older adults (e.g., Gorelik, et al., 2000)? The results of these studies are mixed, suggesting additional elements be considered, such as the quality and outcomes of the experience. This symposium considers the nature, role, and challenges of internship, practicum, and field work experiences from a range of perspectives. First, Dr. Laura Donorfio introduces the components of internships as they relate to workforce development. Next, Dr. Rona Karasik provides a faculty perspective on intergenerational internships. Third, Dr. Phyllis Greenberg and Jodi Danielson offer insight into the agency and preceptor perspective. Forth, Jessica VanderWerf presents a gerontology student’s perspective. Fifth, Camryn Hafner shares the perspective of a student from outside of gerontology – in this case, drama therapy. Finally, moderator and discussant Dr. Marilyn Gugliucci synthesizes the presentation findings and reflects on the promise of gerontological internships to develop professionals both within and outside of gerontology to best meet the demographic reality of the next 30 - 50 years.

